# Novel Therapies and Approaches to Inflammatory Bowel Disease (IBD)

**DOI:** 10.3390/jcm11154374

**Published:** 2022-07-28

**Authors:** Federica Furfaro, Elisa Ragaini, Laurent Peyrin-Biroulet, Silvio Danese

**Affiliations:** 1 Department of Gastroenterology, Istituto di Ricovero e Cura a Carattere Scientifico (IRCCS) Ospedale San Raffaele, 20132 Milan, Italy; furfaro.federica@hsr.it; 2 Department of Biomedical Sciences, Humanitas University, 20090 Milan, Italy; elisa.ragaini@gmail.com; 3 Department of Gastroenterology, Nancy University Hospital, Université de Lorraine, 54500 Vandoeuvre-les-Nancy, France; peyrinbiroulet@gmail.com; 4 University Vita-Salute San Raffaele, 20132 Milan, Italy

Inflammatory bowel diseases (IBDs), divided into two predominant groups, Crohn’s disease (CD) and ulcerative colitis (UC), are chronic relapsing inflammatory diseases of the gastrointestinal tract, resulting from an aberrant immune response to microbes in the gut, in genetically susceptible patients [1].

Many novel therapeutic strategies have been developed in recent decades to induce clinical and endoscopic remission in IBD patients. Drugs that are routinely used to achieve these goals are corticosteroids, aminosalicylates, immunosuppressive and immunomodulator agents [2]. In recent years, biologic therapy has gained increasing attention due to its proven efficacy in achieving clinical remission and mucosal healing and to prevent irreversible structural bowel damage [3,4]. However, there are some limitations in their use related to the development of immunogenicity with a loss of response, primary not response and their invasive route of administration (subcutaneous or intravenous) [5].

Among the currently available biologic agents there are three classes [3]: (I) anti-tumor necrosis factor-alpha (TNF-α) agents, such as infliximab, adalimumab, certolizumab and golimumab, monoclonal antibodies against soluble and transmembrane TNF [6]; (II) anti-integrin agents providing blockade of lymphocytes’ trafficking specifically in the gut, including vedolizumab (anti α4β7 approved for UC and CD) and natalizumab (which targets the α4 subunit in α4β7 and α4β1 integrins, approved for CD) [6]; and (III) anti-interleukin (IL) agents such as ustekinumab, a monoclonal antibody directed to the p40 subunit of IL-12 and IL-23, and risankizumab, a monoclonal antibody targeting the p19 subunit of IL-23. In addition to biologics, small-molecule agents have gained acceptance in the treatment of IBD, including anti-JAK and anti-sphingosine 1 phosphate (S1P) drugs [3].

Many drugs are under evaluation in the field of IBD, and many phase 2 and phase 3 studies are ongoing. Novel agents proposed for IBD treatment are:

- Drugs modulating leukocyte adhesion targeting integrins responsible for homing, to reduce the inflammatory cell infiltrate. For Etrolizumab (anti-β7), there are several phase 3 clinical trials evaluating its efficacy (UC: Hibiscus I and II, Gardenia, Laurel, Hickory; CD: Bergamot) and two open-label extension trials (UC: Cottonwood; CD: Juniper). Its effectiveness is comparable with infliximab to induce clinical response in moderate-to-severe UC patients at 10 and 54 weeks (Gardenia phase 3 study) [7]; however, UC patients treated with etrolizumab do not achieve clinical remission at 62 weeks compared with placebo (Laurel study) [8]. Abrilumab (anti α4β7, AMG-181/MEDI-7183) was evaluated in phase 2 studies in UC, achieving a significant clinical remission rate at week 8 compared with a placebo [9], unfortunately in CD patients, the primary endpoint (clinical remission at week 12) was not met [10]. To date, phase 3 trials for abrilumab have not been registered. Carotegrast (anti α4, AJM300) is an oral drug tested in phase 2 trials for UC, demonstrating significantly higher rates of clinical remission and mucosal healing at 8 weeks compared with the placebo group [11], and there is an ongoing phase 3 trial in UC [12]. Ontamalimab (anti-MAdCAM-1, SHP647, PF-00547659), subcutaneously administered, was evaluated in UC [13] and CD patients [14] in phase 2 trials. In UC patients, data are promising; whereas in CD patients, the primary endpoint (reduction in Crohn’s Disease Activity Index (CDAI) at week 8 or 12) was not achieved when comparing treatment and placebo groups; however, recently published data showed long-term (72 weeks) efficacy and safety of the drug [15]. Finally, PN-943 and PTG-100, two oral anti α4β7 drugs evaluated in UC patients [16], seem promising and there are ongoing trials evaluating both PTG-100 [17] and PN-943 (IDEAL study) [18].
- Janus kinase (JAK) inhibitors target the JAK family, which is composed of four intracellular tyrosine kinases: JAK1, JAK2, JAK3, and nonreceptor tyrosine-protein kinase 2. In UC patients, tofacitinib (anti-JAK1 and -JAK3) was the first approved anti-JAK; however, to reduce the side effects, more selective molecules have been proposed. Phase 3 trials have been completed in UC patients for filgotinib (anti-JAK1) [19] and upadacitinib (anti-JAK1) [20], with good results. Phase 3 trials in CD patient are ongoing or the recruitment status is completed, but final data have not been published for filgotinib [21] and upadacitinib [22]. There are other ongoing phase 2 trials evaluating new molecules such as deucravacitinib (selective TYK2 inhibitor) in UC (Lattice-UC) [23] and in CD patients (Lattice-CD) [24], brepocitinib (dual TYK2/JAK1 inhibitor) in UC [25] and CD patients [26], and PF-06826647 (dual TYK2/JAK2 inhibitor) in UC patients [27], with promising results presented at international congresses.
- Sphingosine-1-phosphate subtype 1 (S1P1) receptor modulators, such as Ozanimod (RPC1063) and Etrasimod. Ozanimod is more effective than placebos as induction and maintenance therapy in patients with moderately to severely active UC, as showed in a recently published phase 3 study [28] and in phase 2 study in CD patients [29]. There are ongoing phase 3 studies in CD [30,31,32]. Etrasimod is more effective than a placebo in inducing clinical and endoscopic improvements in UC patients, and demonstrated a favorable safety profile [33].
- ABX464 is a potent up-regulator of miR-124, an anti-inflammatory microRNA; it is more effective than a placebo in achieving endoscopic improvements and clinical responses in UC patients [34]. Currently, there is an ongoing phase 2 trial assessing the efficacy of this agent [35].

The relevant number of clinical trials evaluating the efficacy of newly developed drugs testifies the increasing importance that target therapy has among the current treatment options for IBD. Medical care is developing towards a less invasive and more personalized approach, and the advent of the new aforementioned therapies for IBD furthers research in this direction. First, by increasing the therapeutic options, the number of patients requiring invasive treatment procedures (i.e., surgery) will decrease, leading to less invasive treatment protocols; second, by expanding the therapeutic armamentarium available to IBD physicians, the standard of care will start to shift towards a truly personalized, patient-centered approach.
